# Comparative Analysis of P2X1, P2X2, P2X3, and P2X4 Receptor Subunits in Rat Nodose Ganglion Neurons

**DOI:** 10.1371/journal.pone.0096699

**Published:** 2014-05-05

**Authors:** Lizhao Wang, Dan Feng, Huanhuan Yan, Zhongping Wang, Lei Pei

**Affiliations:** 1 Key Laboratory of Neurological Diseases of Ministry of Education, Tongji Medical College, Huazhong University of Science and Technology, Wuhan, China; 2 CNS Pharmacology & Ion Channel Group Shanghai Chempartner, Shanghai, China; 3 Department of Pain Clinic, Institute of Anesthesia and Critical Care, Union Hospital, Tongji Medical College, Huazhong University of Science and Technology, Wuhan, China; 4 Department of Physiology and Pathophysiology, Jiujiang University, Jiujiang, China; Dalhousie University, Canada

## Abstract

Nodose ganglion (NG) neurons are visceral primary sensory neurons. The transmission and regulation of visceral sensation is mediated mainly by the P2X purinoceptor (P2X receptor). Although the characteristics of different P2X receptor subunits in the NG have been studied previously, comprehensive analyses have not been performed. In this study, we used immunohistochemistry, immunocytochemistry, and whole cell patch clamp techniques to compare the expression and function of P2X1, P2X2, P2X3, and P2X4 receptor subunits in adult rat NG neurons. Polyclonal antibodies against the four P2X subunits labeled different subpopulations of NG neurons. P2X1 and P2X3 were expressed mainly in small-to-medium sized NG neurons, whereas P2X2 and P2X4 were located mostly in medium- and larger-sized NG neurons. Over 36% of NG neurons were P2X3 positive, which was higher than the other three P2X subunits. In addition, different types of currents were recorded from neurons expressing different P2X subunits. The fast type of ATP current was recorded from neurons containing P2X1–4 subunits, the intermediate type of current was recorded from neurons containing the P2X1, P2X3, and P2X4 subunits, the slow type was recorded from neurons expressing P2X1–3, and/or P2X4 subunits, whereas the very slow type was recorded from neurons containing the P2X2 and P2X3 subunits. These comparative results provide an anatomical verification of the different subunits in NG neurons, and offer direct support for the idea that various functional NG populations have distinct responses to ATP, which might be in part due to the different expression profiles of diverse P2X subunits.

## Introduction

Placode-derived general visceral afferent neurons of the nodose ganglion (NG) transmit visceral sensory information from specialized sensory endings of the vagus nerve and its branches to the nucleus of the solitary tract [1]. These neurons are critical for relaying various endogenous and exogenous stimuli. Multiple neurotransmitters and neuromodulators are associated with NG neurons, which also contain a variety of receptors that respond to transmitters, inflammatory mediators, and neurotrophic factors [2]–[3].

Adenosine 5′-triphosphate (ATP), an excitatory neurotransmitter, acts on P2X purinoceptors (P2X receptor) that are formed by the assembly of three of the seven subunits, P2X1–7, to induce inward, non-selective cation currents (I_ATP_) [4]–[6]. Studies on the localization of the P2X receptor have been performed using *in situ* hybridization, and polyclonal antibodies against P2X receptor subunits have been developed and used to identify P2X receptors in peripheral and central nervous tissues [7]. ATP-activated currents are classified under different categories according to cell size and electrophysiological properties [8]–[9]. However, quantitatively comparative studies on the expression patterns of P2X receptors in NG neurons are rare, and it remains unclear whether these receptors are expressed in the same patterns within ganglia.

In the present study, we investigated the expression patterns and functions of the four critical P2X receptor subunits (P2X1, P2X2, P2X3, and P2X4) in NG neurons, and performed a comparative analysis at both the cellular and tissue levels. Our findings provide anatomical evidence for a possible relationship between responses to ATP and different P2X receptor subunits in NG neurons.

## Results

### P2X1, P2X2, P2X3, and P2X4 Expression in the NG

**Figure 1:**
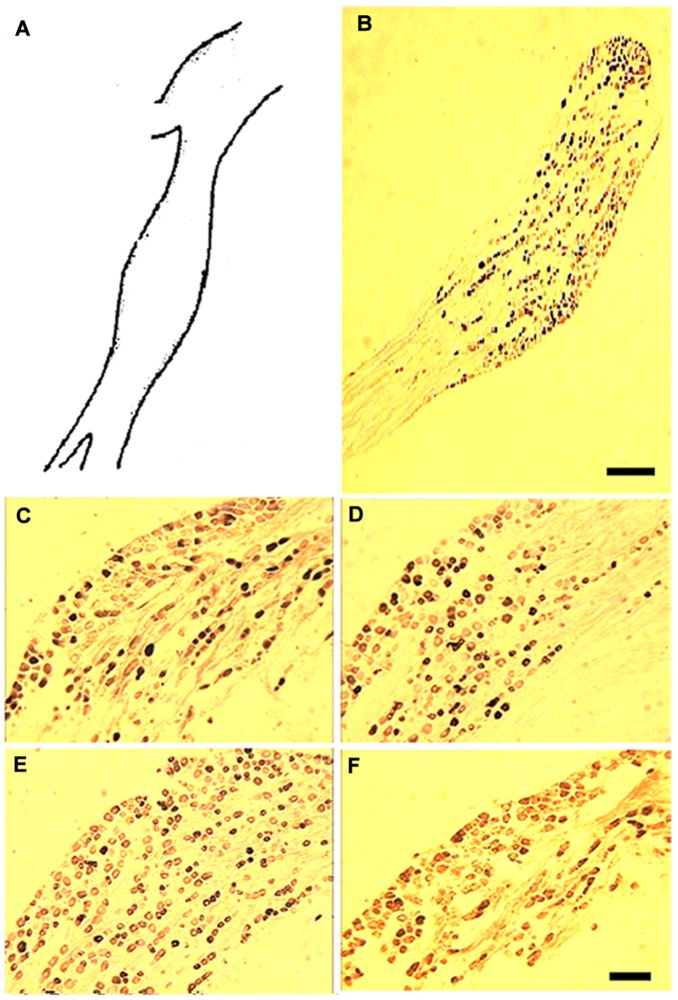
Distribution and expression of P2X1–4 subunits in NG tissue. (A) A schematic diagram of the maximum cross section of a rat nodose ganglion (A, corresponding to panel B). (B) Representative graph of the distribution of P2X receptor-positive cells throughout the whole nodose ganglion section under a 20× light microscopic field. (C–F) Immunohistochemical staining using polyclonal antibodies against P2X1 (C), P2X2 (D), P2X3 (E), and P2X4 (F); 100× magnification. Scale bars in B = 500 µm and F = 100 µm.

The sensory root of the vagus nerve extended from the dorsolateral medulla oblongata, ran through the cranial cavity, and emerged at the cervical region of the jugular foramen. In the cranial cavity, the vagus nerve includes the nodose ganglion (∼1 mm long, Fig. 1A), which is distal to the jugular ganglion along the internal jugular vein at the jugular foramen. Most neurons throughout the NG exhibited positive staining for P2X subunits. A typical example is illustrated in Fig. 1B. Based on the staining intensity, two types of neurons were observed: strongly stained neurons with cytoplasmic immunoreactivity, and lightly stained neurons with weak staining that was restricted to the plasma membrane and cytoplasm (Fig. 1C–F). The strong cytoplasmic staining disappeared in the NG sections, whereas the weak membrane and cytoplasmic staining remained unchanged. This suggests that the light staining might be non-specific; therefore, only strongly-stained neurons were analyzed in subsequent studies.

### Analyzing the Size of P2X1-, P2X2-, P2X3-, and P2X4-positive Neurons in the NG

**Figure 2:**
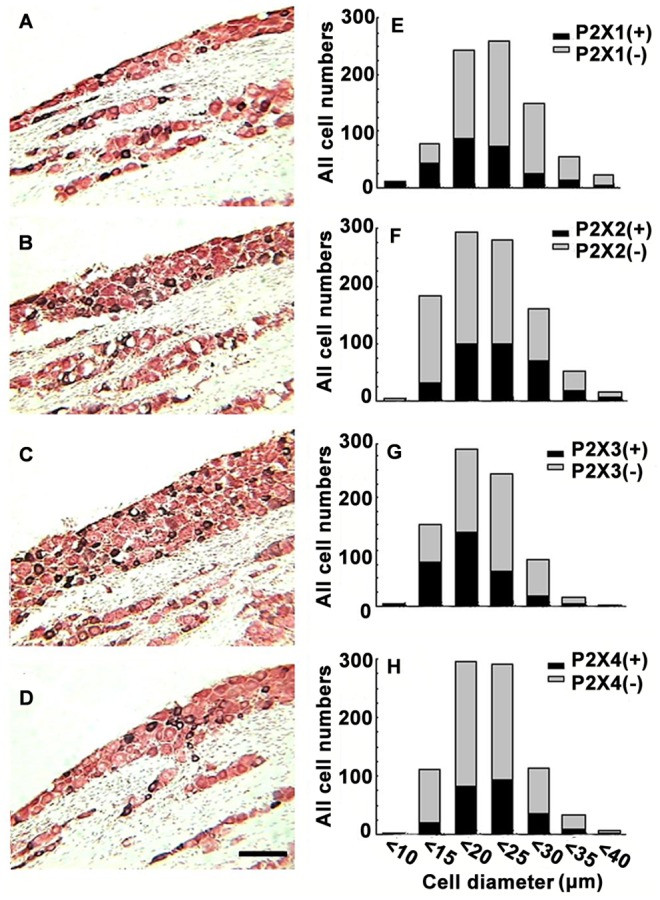
The frequency distribution of the area-size of P2X1–4 subunit-positive neurons. (A), (B), (C), and (D) show P2X–IR sections that were counterstained with neutral red to precisely calculate the number of P2X1, P2X2, P2X3, and P2X4-positive neurons, respectively (using black positive cells compared with red negative cells). Scale bar in (D)  = 100 µm. (E), (F), (G), and (H) represent the frequency distribution of the area-size of P2X1, P2X2, P2X3, and P2X4-IR, respectively.

To accurately count, measure, and analyze the cell size (diameter) and distribution of P2X-positive neurons in the NG, immunoreactive sections were counter-stained with 1% neutral red. As shown in Fig. 2, black colored cells were positive, whereas red colored cells were negative (Fig. 2A–D). Only cells with a nucleus were included and measured. Neurons in the NG expressed all four P2X receptor subunits (P2X1, P2X2, P2X3, and P2X4). The immunoreactive intensities of the four P2X subunits were comparable (Fig. 2A–D). The P2X1-immunoreactive (IR) neurons in the NG were round or oval cells sized 3.6–32.5 µm (Fig. 2A, E). Immunohistochemistry for P2X2 showed that P2X2-IR neurons were located throughout the NG, and ranged from 6.9 to 34.7 µm (Fig. 2B, F). P2X3-IR neurons were also round or oval cells sized 4.2–33.6 µm (Fig. 2C, G). P2X4-IR neurons in the NG were sized 7.1–34.5 µm, which was slightly larger than P2X3-IR neurons (Fig. 2D, H). Therefore, P2X1- and P2X3-IR neurons were mostly small, with some medium sized (13.6±2.9 and 15.1±3.2 µm, respectively, mean ± SEM). In contrast, P2X2 and P2X4-IR neurons were mainly medium, and some larger sized (21.8±2.4 and 23.4±1.3 µm, respectively, mean ± SEM). No immunoreactive neurons were detected in the NG sections processed without primary antibodies.

### Ratio of P2X1, P2X2, P2X3, and P2X4 Subunits in all NG Neurons

**Figure 3:**
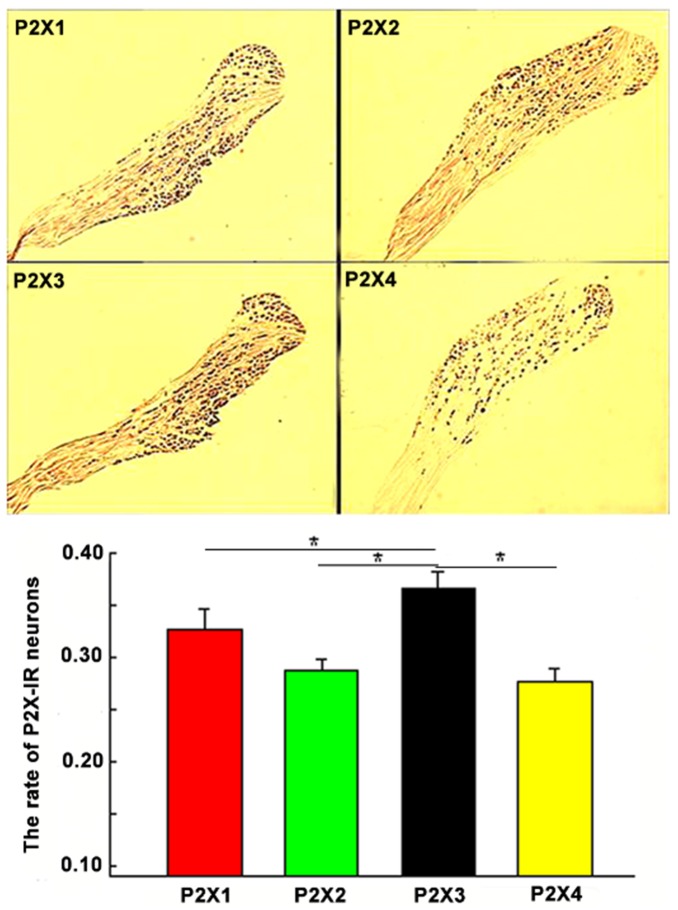
The proportion of P2X1–4 subunit-expressing neurons compared with the total number of NG neurons. The upper panel shows immunohistochemical staining for P2X1–4 subunits in the maximum cross section of the NG tissues. The lower panel shows that P2X3-IR was the most prevalent neuronal subtype, following by P2X2-IR; P2X4-IR had the lowest prevalence. *p<0.05.

To precisely calculate the ratio of P2X subunit-expressing neurons within the NG, immunohistochemical staining of NG tissue sections was performed in primary cultured NG neurons using P2X1, P2X2, P2X3, and P2X4 polyclonal antibodies. The percentage of P2X1, 2-, 3- and 4-stained neurons was 32.68±1.87, 28.71±1.12, 36.62±1.56, and 27.70±1.16%, respectively (Fig. 3). Among the four P2X receptor subtypes, the expression of P2X3 was the highest. The percentage of P2X1 in the NG was lower than P2X3, but higher than P2X2 or P2X4 (*n* = 5, *p*<0.05, analysis of variance, [ANOVA]). The P2X4 subunit was less prevalent. However, there were no significant differences among the ratios of P2X1, P2X2, and P2X4 (*n* = 5, *p*>0.05, ANOVA).

**Figure 4:**
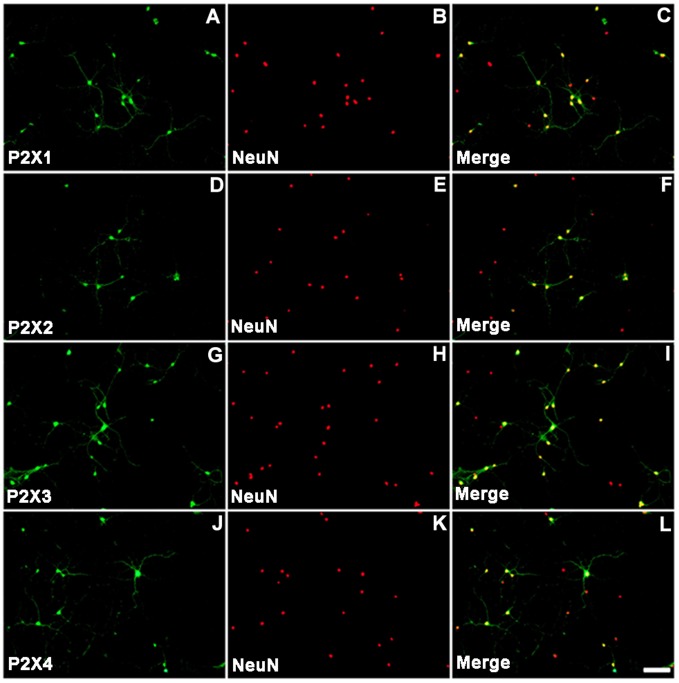
Immunofluorescent staining of cultured NG neurons. NG neurons were stained using antibodies against P2X1, P2X2, P2X3, and P2X4 receptor subunits (A, D, G, and J, respectively). The nuclei of cultured NG neurons were stained with antibodies against NeuN (B, E, H, K). Merged images (C, F, I, L) representing co-staining of P2X receptor subunits and NeuN are shown. The scale bar shown in L is representative of all images, and represents 50 µm.

Primary cultured NG neurons were stained with anti-NeuN antibodies to discriminate neurons from other cells. Therefore, P2X and NeuN double-stained cells were P2X-positive neurons. Figures 4A, D, G, and J show positive staining for the P2X1, P2X2, P2X3, and P2X4 subunits in NG neurons on *in vitro* day 10, respectively. Similarly, Figures 4B, E, H, and K show positive staining in the nucleus of primary cultured NG neurons, whereas figures 4C, F, I, and L show co-staining of P2X and NeuN. These data suggest that NG cultured neurons contain significant concentrations of the P2X subunits, which is consistent with the results obtained from tissue staining experiments (Figs. 1, 2, and 3).

### Co-localization of the P2X Subunits in the NG

**Figure 5:**
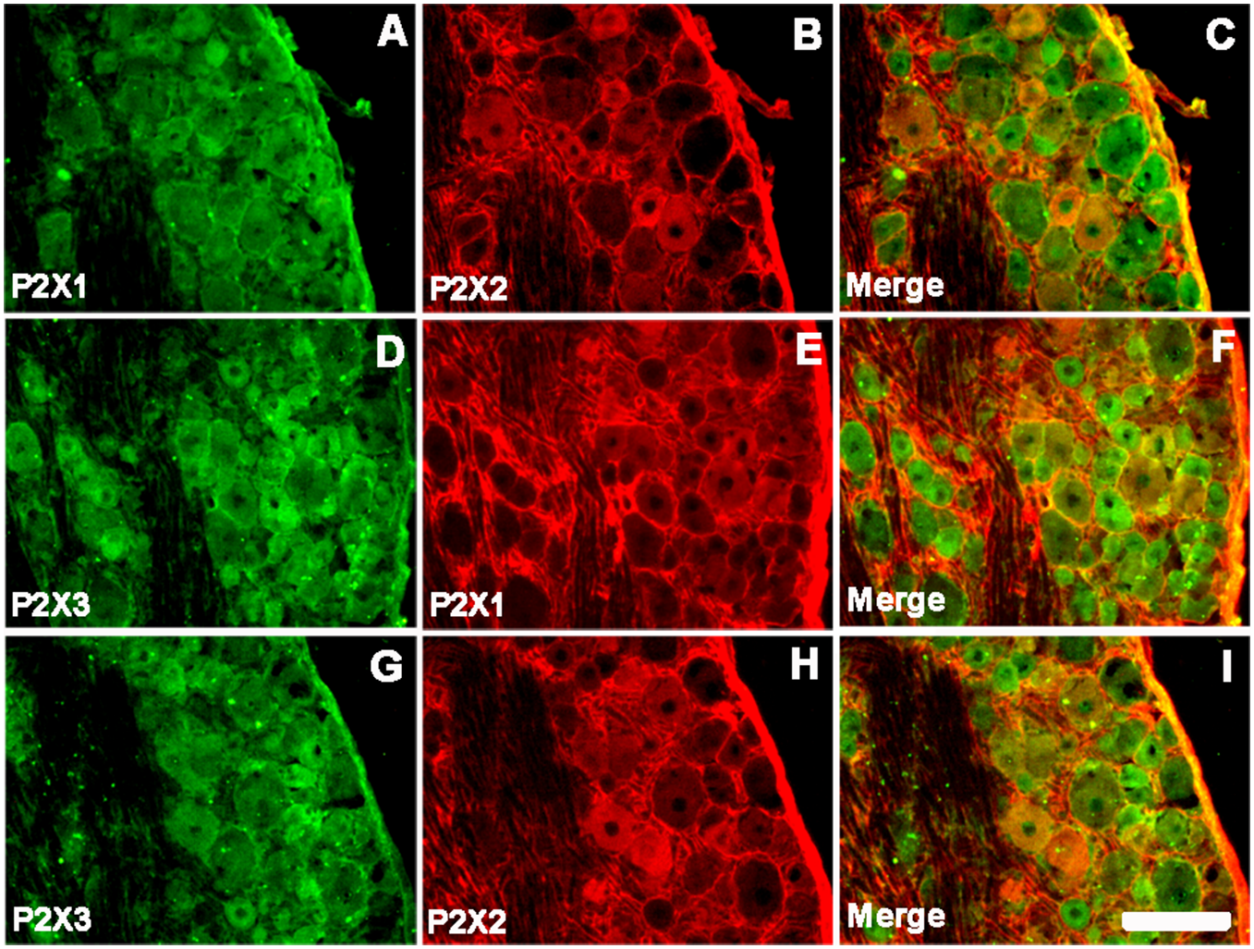
Co-localizations of two P2X subunits in nodose ganglia. (A–C) Immunoreactivity of P2X1 and P2X2 (A–C), P2X1 and P2X3 (D–F), and P2X2 and P2X3 (G–I) subunits in the NG. Scale bars = 50 µm.

The P2X receptors in the NG sensory neurons are heteropolymers of P2X1 and P2X2 (P2X1/2) [10], P2X1 and P2X3 (P2X1/3), and P2X2 and P2X3 (P2X2/3) [11]. In order to confirm these reports, we next performed double immunofluorescent staining of NG tissue sections. The results demonstrated that P2X1-IR was very often co-expressed with P2X2-IR; the P2X3-IR profiles were also P2X1 receptor immunoreactive. Meanwhile, P2X3-IR co-stained much stronger with P2X2-IR than with P2X1-IR (Fig. 5A–I), suggesting that the main subunits expressed by sensory neurons were P2X3 and P2X2. In addition, P2X3-IR NG neurons co-localized with P2X4-IR neurons, and P2X4 was also coexpressed with P2X1 and P2X2 (data not shown). These results suggest that native receptor subunits might form homo- and heteropolymers to respond to adenosine triphosphate (ATP).

### ATP Induced Four Types of Currents in Different Sized NG Neurons

**Figure 6:**
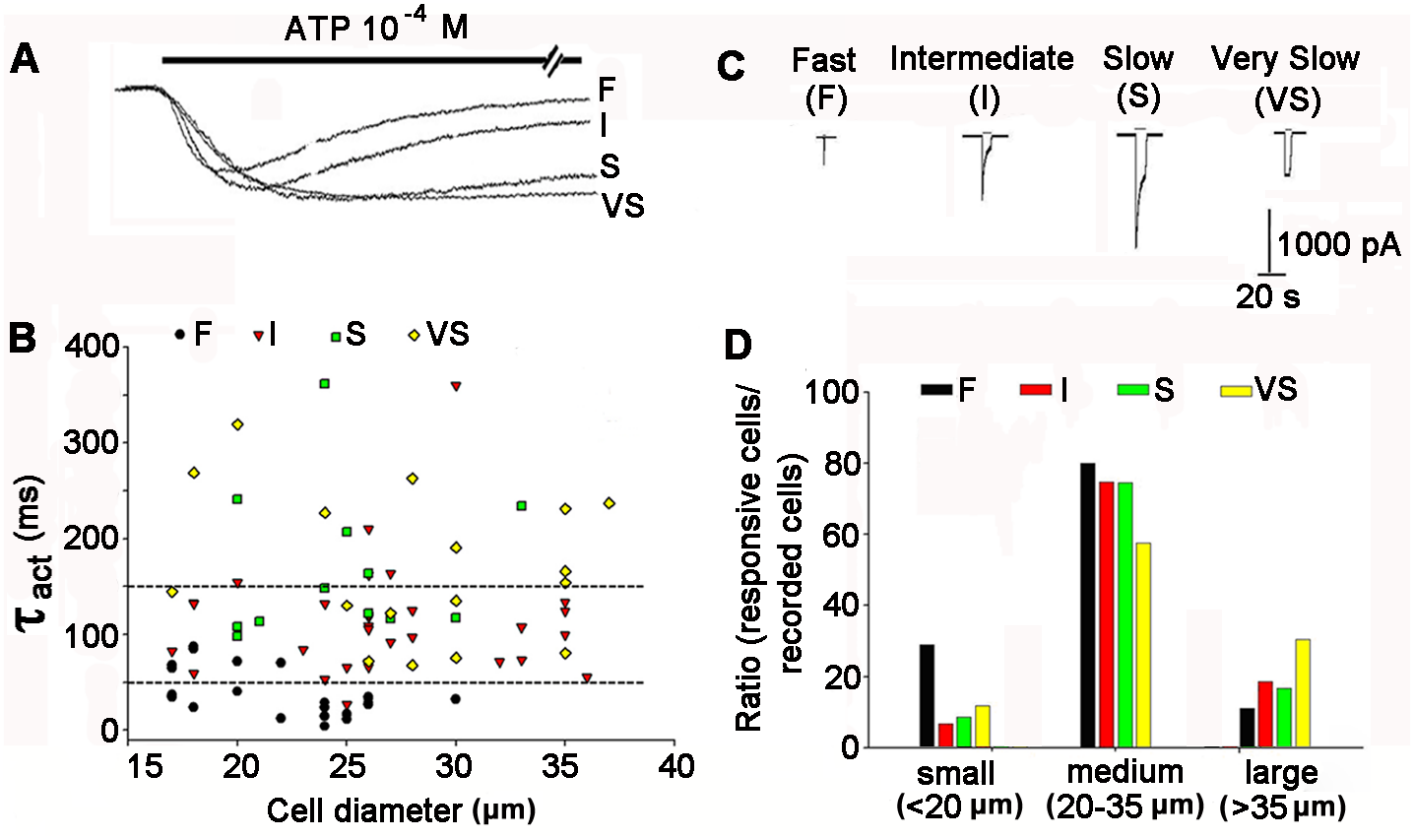
Four types of I_ATP_s and their relationship with NG neuron diameter. (A) The currents activated by ATP (10^−4^ M) in NG were characterized according to fast (F), intermediate (I), slow (S), and very slow (VS) kinetics with an expanded time axis. The horizontal bar above the traces indicates the application of ATP. (B) The 10–90% rising time (ordinate) of different types of ATP (10^−4^ M)-activated currents (black circles  =  type F, red triangles  =  type I, green squares  =  for type S, and yellow diamonds  =  type VS I_ATP_s) against neuron diameter. Each point in the graph represents a single cell. The correlation coefficient was 0.66 (*p*<0.05). (C) Representative traces of type F, I, S, and VS ATP (10^−4^ M)-activated currents. (D) Comparisons of the absolute ratios of the four types of I_ATP_s in different subpopulations (small, medium, and large sizes) of NG neurons. Specifically, F type: small (13/54, 28.9%), medium (36/54, 80.0%), and large (5/45, 11.1%); I type: small (5/75, 6.7%), medium (56/75, 74.7%), and large (14/75, 18.6%); S type: small (11/126, 8.7%), medium (94/126, 74.6%), and large (21/126, 16.7%); VS type: small (7/59, 11.9%), medium (34/59, 57.6%), and large (18/59, 30.5%).

ATP was applied externally to induce P2X currents to investigate the functions of P2X receptor subunits in NG neurons. A total of 444 NG neurons were recorded. Most NG neurons (312/444, 70.3%) were sensitive to ATP (3×10^−5^ to 3×10^−3^ M), whereas the others had no response (132/444, 29.7%). Among the ATP-responsive NG neurons, the I_ATP_ current traces were all inward, but with different rates of rise and decay (Fig. 6A and C). Based on the kinetic features, four types of distinct ATP induced currents (I_ATP_s) were classified: F (fast, fast activation and desensitization, 54/444, 12.2%), I (intermediate, fast activation, but slow desensitization, 73/444, 16.4%), S (slow, slow activation and desensitization, 126/444, 28.4%), and VS type (very slow, slow activation and very slow desensitization, 59/444, 13.3%). Fig. 6B shows the 10–90% rising time (ordinate) of the four types of ATP (10^−4^ M)-activated currents (black circles = F, red triangles = I, green squares = S, and yellow diamonds = VS I_ATP_s) plotted against neuron diameter.

In addition, we measured the diameters of the NG neurons using a whole cell patch clamp, and found that different sized NG neurons had different types of I_ATP_. F type currents were recorded mostly on small- (<20 µm) and medium-sized (20∼35 µm) neurons, I and S type currents were induced mostly on medium sized neurons, and VS type currents were mainly activated on medium and large sized (>35 µm) neurons (Fig. 6D, Table 1).

**Table 1:** Relationship between the numbers and sizes of four types of I_ATP_ NG neurons.

Types of I_ATP_	Cell sizes		
	Small (<20 µm)	Medium (20∼35 µm)	Large (>35 µm)
Fast	44/54 (81.5%)	10/54 (18.5%)	0
Intermediate	19/75 (25.3%)	53/75 (70.7%)	3/75 (4.0%)
Slow	39/126 (30.1%)	83/126 (65.9%)	4/126 (3.0%)
Very Slow	1/59 (1.7%)	33/59 (55.9%)	25/59 (42.4%)

### Concentration–response Relationships of the Four Types of I_ATP_

**Figure 7:**
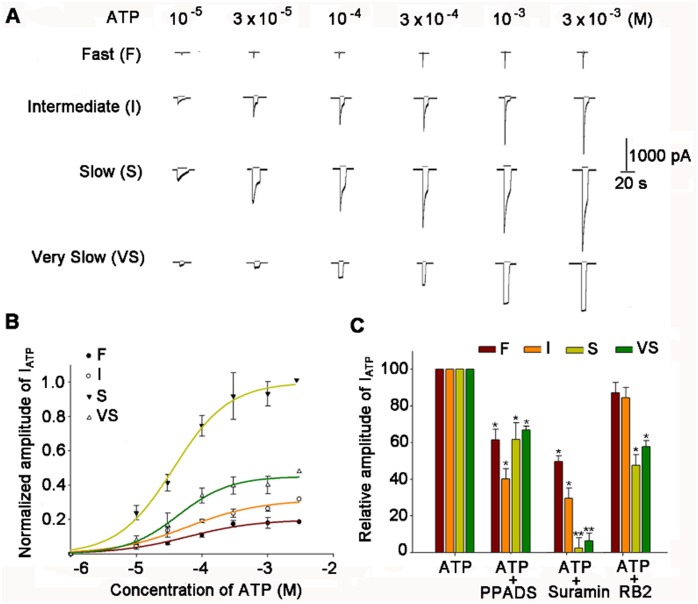
Concentration-response relationships and the efficacy order of P2X receptor antagonists on I_ATP_s. (A) Sequential current traces of F, I, S, and VS ATP-activated currents recorded from rat NG neurons in response to different concentrations of ATP (from 10^−5^ to 3×10^−3^ M). Current traces of each type were obtained from the same neuron. (B) The dose-response curves for each type of I_ATP_s. Each point represents the means ± SEM of 10–15 neurons. All ATP-induced currents were normalized to the response induced by 3×10^−3^ M ATP in each type. The holding potential was set at −60 mV. The data for ATP were a good fit to the Hill equation I = I_max_/[1+ (EC50/C) n], where C is the concentration of ATP, I is the normalized amplitude of I_ATP_, and EC50 is the concentration of ATP for the half maximal current response. (C) The efficacy order of the inhibitory effects of P2X receptor antagonists on four distinct I_ATP_s. The columns in the bar graph show the inhibitory effects of the P2X receptor antagonists: PPADS (10^−4^M), suramin (10^−4^ M), and RB2 (10^−4^ M). F-type, suramin >PPADS > RB2; I-type, suramin > PPADS > RB2; S-type, suramin > RB2> PPADS; VS-type: suramin > RB2> PPADS. *p<0.05, **p<0.01.

The amplitude of the four types of ATP-activated current was concentration-dependent. The concentration–response relationships of the four types of I_ATP_ activated by 10^−5^ to 3×10^−3^ M ATP are shown in Fig. 7A. These data demonstrate that: (1) the threshold and saturation concentrations for the four types of I_ATP_ were essentially identical, with values of 10^−5^ and 3×10^−3 ^mol/L, respectively; (2) the EC50 values of the four types of I_ATP_ were close, being 5.89×10^−5^ (F), 5.92×10^−5^ (I), 3.67×10^−5^ (S), and 4.34×10^−5^ (VS) M (n = 6–10, *p*>0.05), with Hill coefficients of 0.81 (F), 0.78 (I), 1.03 (S), and 1.11 (VS), respectively (*p*>0.05) (Fig. 7B).

### Efficacy of P2X Receptor Antagonists on the Four Types of I_ATP_

Next, several different P2X receptor antagonists, including reactive blue 2 (RB2), suramin, and pyridoxal-phosphate-6-azophenyl-2′, 4′-disulfonic acid (PPADS), were used to assess the effects of P2X receptor antagonists on each I_ATP_s in NG neurons. All four types (F, I, S, and VS) of I_ATP_ (10^−4^ M) were inhibited by 1 min pre-treatment with 10^−4^ M PPADS (63.6±5.2, 42.5±4.3, 64.8±7.3, and 68.2±1.6%, respectively), RB2 (51.3±2.6, 27.1±3.5, 2.6±3.3, and 4.5±2.1%, respectively), and suramin (87.2±3.6, 85.4±4.2, 45.5±4.1, and 58.7±2.6%, respectively) (*p*<0.05). However, the inhibitory effects of 10^−4^ M suramin on I_ATP_ (10^−4^ M) were much stronger than those of 10^−4^ M PPADS or RB2. Specifically, for F and I type I_ATP_ the efficacy order was suramin >PPADS > RB2, compared with suramin > RB2>PPADS for S and VS type (Fig. 7C).

### Association between the Four Types of I_ATP_ and the Expression of P2X1−4 Subunits

**Figure 8:**
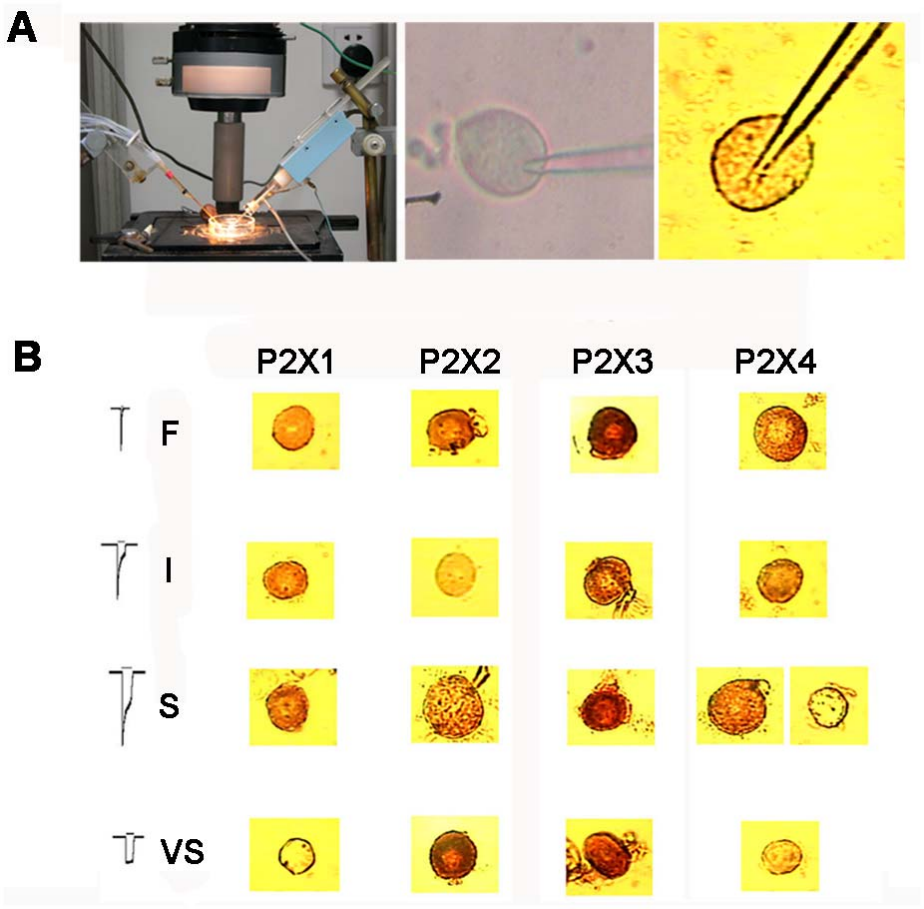
Relevance of the P2X1–4 subunits on the four types of I_ATP_. (A) Schematic view of the setup for the whole cell patch clamp and a representative image of a recorded cell under the phase contrast microscope and immunohistochemistry. (B) Immunohistochemistry revealed positive or negative staining for P2X1–4 subunits, which correlated with the type of I_ATP_ and cell size. The samples in each row were from four different neurons that responded to ATP with different types of ATP-activated current. P2X3 staining was positive in all four types of I_ATP_ neurons. P2X1 was positive in F, I, and S I_ATP_s, but negative in VS. P2X2 staining was only absent in neurons with type I I_ATP_, and P2X4 was positive in neurons with type F, I, and some S I_ATP_s.

Different phenotypes of ATP-activated currents could be attributed to different subunit assemblies. To provide direct evidence for this hypothesis, we investigated the relevance of the four types of I_ATP_ on the expression of P2X1−4 subunits by combining the whole-cell patch clamp technique with single cell immunohistochemistry. As described in the Materials and Methods and Fig. 8A, individual cell was harvested using a pipette tip after patch clamp recording had finishing, and immunohistochemistry was then performed (Fig. 8B). ATP-responsive cells with type F I_ATP_ exhibited positive staining for P2X1, P2X2, P2X3, and P2X4. ATP-responsive cells with type I I_ATP_ demonstrated positive staining for P2X1, P2X3, and P2X4, but negative staining for P2X2. In contrast, cells responsive to ATP with type S I_ATP_ showed positive staining for P2X1, P2X2, and P2X3, but only a small number of cells were P2X4 positive. Finally, ATP-responsive cells with type VS I_ATP_ stained positive for P2X2 and P2X3, but negative for P2X1 and P2X4. The correlation between I_ATP_ type (F, I, S, and VS) and underlying subunit composition is summarized in Tables 1 and 2.

**Table 2:** Relevance of P2X1–4 subunits staining with four types of I_ATP_s.

	P2X1	P2X2	P2X3	P2X4
Fast	+ (5)	+ (6)	+ (8)	+ (4)
Intermediate	+ (4)	− (3)	+ (5)	+ (5)
Slow	+ (4)	+ (4)	+ (3)	+ (5)/− (3)
Very Slow	− (5)	+ (3)	+ (4)	− (4)

+ stands for positive staining, − stands for negative staining.

## Discussion

In this study, we investigated the characteristics of P2X1, P2X2, P2X3, and P2X4 receptor subunits in NG neurons using immunohistochemistry, immunocytochemistry, and whole cell patch clamps. Our data demonstrate that, 1) NG neurons abundantly express P2X1, P2X2, P2X3, and P2X4 subunits. 2) P2X1 and P2X3 subunits are mainly expressed in small and medium-sized NG neurons, whereas P2X2 and P2X4 subunits are dominantly expressed in large NG neurons. 3) The expression ratio of P2X3 receptors was higher than the other three receptor subunits, followed by P2X1, P2X2, and P2X4. 4) P2X1 was co-expressed with P2X2 and P2X3, P2X3-IR co-localized with P2X2-IR and P2X4-IR, whereas P2X4 was detected with P2X1 and P2X2 separately in NG neurons. 5) The I_ATP_s recorded from rat NG neurons could be classified into four types (F, I, S, and VS), and the type distinction of I_ATP_s was associated with different P2X receptor subunit expression. Our results suggest that the P2X receptor subunit distribution varies in different subpopulations of NG neurons, suggesting that diversity of receptor subunits assemblies might exist.

Since the original cloning of the P2X3 subunit from NG [12]–[13], more attention has been paid to this ganglion because of the possible involvement of P2X receptor subunits in nociception [14]. Our present study is consistent with a previous reporting that *P2X1*, *P2X2*, *P2X3*, and *P2X4* mRNAs are abundant in the NG [15]. In addition, the parallel expression of the P2X1–4 subunits in both NG tissue section and cultured NG neurons suggests consistent expression profiles of P2X receptor subunits in different subpopulations of NG neurons, which might play roles in mediating ATP signals. Small diameter neurons, which have thin unmyelinated C-fibers, conduct nociceptive stimuli [16]–[19]. While medium and large sized neurons likely have myelinated Aδ and Aβ fibers respectively, which conduct mechanosensory stimuli [20]. Our results demonstrate that small sized NG neurons express high levels of P2X3 and P2X1 receptor subunits, whereas medium and large sized NG neurons contain abundant P2X2 and P2X4 receptor subunits. This suggests that different P2X receptor subunits in different subpopulations of NG neurons might mediate different types of nociceptive sensation. This is consistent with a previous study, where P2X receptor subunits were expressed in NG, Trigeminal Ganglion (TG), and Dorsal Root Ganglion (DRG) sensory neurons, and participated in the transmission of various nociceptive signals [21].

In this study, four types of I_ATP_s recorded from rat NG neurons were defined based on the kinetics of the currents: types F, I, S, and VS. We kinetically used the 10–90% rising time (R10–90) as an indication, which was positively correlated with the size of NG neurons, and observed overlap between types F, I, and S. These results are consistent with evidence that different responses to ATP in the same populations of NG neurons result from different P2X receptor subunit assemblies [22]–[23]. In addition, dose-response curves suggested that P2X subunits have the same threshold values, and that their EC50 values are close. This suggests that the four types of I_ATP_s mediated by the P2X receptor subunits have similar affinities for ATP. Previous study reported that P2X receptor antagonists exhibited slow onset kinetics [24]. We applied the P2X receptor antagonists RB-2, PPADS, and suramin and found that types F and I I_ATP_s were less sensitive to RB-2, types F, S, and VS I_ATP_s were less sensitive to PPADs, and types F and I I_ATP_s were less sensitive to suramin. These results suggest that the affinity and efficacy of these antagonists to P2X receptors might depend on the different assemblies of P2X receptor subunits. Here, only one time point (1 min pre-treatment) and a single concentration (10^−4^ M) of the applied drugs were used to assess the blocking effects. Therefore, additional studies are needed to further clarify the role of P2X receptor antagonists on I_ATP_s in NG neurons.

The expressing of P2X1 or P2X3 subunits exhibited fast activation and rapid desensitizing currents, whereas P2X2 or P2X4 expressed alone yielded sustained ATP-activated currents. However, the co-expression of P2X2 and P2X3 resulted in slow, sustained I_ATP_, whereas P2X3 and P2X4 displayed an I_ATP_ of desensitization with two components of rapid, and slow sustained I_ATP_ [15]. In addition, the expression of P2X3 alone exhibited fast desensitizing I_ATP_, but that the co-expression of P2X2 and P2X3 yielded mixed fast and slow desensitizing I_ATP_ [25]–[26]. Although we did not assess the contribution of P2X5, P2X6, and P2X7 to the features of I_ATP_s, previous studies demonstrated that the expression of cloned P2X5 resulted in a mixed desensitizing I_ATP_ with two components, whereas P2X6 exhibited slow desensitizing I_ATP_, and P2X7 was not found in NG neurons [27]. Boue-Grabot *et al.* correlated molecular structure with the function of different types of P2X receptors, and suggested that P2X1 and P2X3 formed rapid desensitizing heteromeric/homomeric receptors [28], whereas P2X2, P2X4, and P2X5, P2X2 and P2X3, P2X1 and P2X5, and P2X4 and P2X6 homomeric or heteromeric receptors desensitized at a low to moderate rate [29].

By combining a whole cell patch clamp technique with *in situ* immunocytochemistry, we offered direct and convincing evidence that the configurations of I_ATP_s correlated with the composition of P2X1–4 subunits and cell size. These results also confirm previous speculation that small- and medium-sized neurons express P2X1 and/or P2X3 subunits with fast activated and rapid desensitized currents, while medium- and large-sized neurons express P2X2 or P2X4 subunits with fast activated and slowly desensitized currents [30]. However, it should be emphasized that we obtained only limited data from single staining since different P2X subunit assemblies could not be reflected using such procedures. Therefore, double or triple staining of recorded cells should be performed for further investigation.

### Conclusions

Taken together, the present data provide a comprehensive analysis of P2X receptor subunits (P2X1, P2X2, P2X3 and P2X4) in different subpopulations (small-, medium-, and large-sized) of NG neurons. We demonstrated that the type distinction of I_ATP_ was associated with cell size and P2X receptor subunits: small- and medium-sized NG neurons express mainly P2X1 and/or P2X3 subunits with fast activated and rapid desensitized current (type F and I I_APT_s), whereas medium- and large-sized neurons mainly express P2X2 or P2X4 subunits with fast activated and slow desensitized currents (type S and VS I_ATP_s).

## Materials and Methods

### Animals

Male adult Sprague-Dawley rats weighing 150 g were used for immunostaining studies. Animals at postnatal day 0–1 were used for the primary culturing of NG neurons. Rats weighing ∼150 g were used for the acute isolation of NG neurons and the subsequent patch clamp recording studies. All animal experimental procedures were reviewed and approved by the Animal Use and Care Committee at the Huazhong University of Science and Technology, and were performed in accordance with the National Institutes of Health guidelines on animal care.

### Immunohistochemistry and Double Immunofluorescent Staining

Adult male rats (*n* = 5) were anesthetized using chloral hydrate (1.2 g/kg i.p.) and perfused transcardially with heparinized saline followed by 4% paraformaldehyde in phosphate buffered saline (PBS; pH 7.4). After perfusion, the NG tissues were removed, kept in the same fixative for 6 h, and then cryoprotected in 30% sucrose in 0.1 mol/L PB (phosphate buffer) (4°C) overnight. Sections (20 µm) were then cut using a cryostat, collected sequentially in four vials (with a minimum separation of 80 µm between sections), and immersed in 0.01 mol/L PBS (pH 7.4). As described previously [31], immunohistochemical staining for P2X1, P2X2, P2X3, and P2X4 was performed using the avidin-biotin-horseradish peroxidase complex (ABC) detection method (Vector Laboratories, Burlingame, CA). Sections were incubated with rabbit anti-P2X1, P2X2, P2X3, and P2X4 polyclonal antibodies (1∶1000, Cell Signaling Technology, USA) for 24 h at 4°C in a humid atmosphere. After rinsing with PBS, the sections were incubated with biotinylated goat anti-rabbit IgG (1∶200) for 1 h in a humid atmosphere at room temperature, followed by ABC complex (1∶800) for 1 h. All antisera were diluted in 0.01 mol/L PBS containing 0.3% Triton X-100. The sections were then stained for 5 min in a solution containing 0.05% 3, 3′-diaminobenzidine tetrahydrochloride (DAB, Sigma-Aldrich, St. Louis, MO), and activated with 0.01% H_2_O_2_. Finally, the sections were mounted on gelatin-coated glass slides, air-dried, dehydrated in a graded series of alcohols, cleared in xylene, and cover-slipped with resin (Shanghai Resin Factory Co., LTD, Shanghai, China). Control sections were incubated without the primary antibody, and no staining was observed. Double immunofluorescence staining of ganglion sections was described previously [32]. Mounted NG sections were thawed at room temperature, and indirect immunofluorescence was used to detect P2X1, P2X2, P2X3, and P2X4. Dual-labeling immunofluorescence was used to analyze the co-localization of two P2X subunits. Samples were blocked with donkey serum for 1 h. Next, samples were incubated with rabbit anti-P2X1, mouse anti-P2X2, goat anti-P2X3, and chicken anti-P2X4 polyclonal antibodies (1∶200, Santa Cruz Biotechnology, Santa Cruz, CA) for 48 h. FITC- and TRITC- conjugated secondary antibodies were then incubated for 1 h.

### Primary Culture of Nodose Ganglion Neurons

Rat NG neurons were cultured as previously described [33]–[34]. Briefly, the NG was dissected and dissociated from postnatal day 1 pups that were sacrificed by cervical dislocation under sterile conditions. The ganglia were then incubated for 15 min at 37°C in D-Hank’s balanced salt solution (without Ca^2+^ and Mg^2+^) with collagenase (type I, 1 mg/ml; Sigma). The cell suspension was then washed once with L-15 growth medium containing 10% horse serum. The pellet was resuspended and centrifuged through a Percoll (Pharmacia) gradient (35%) to separate neurons from non-neuronal cells. The neuronal suspension was then washed twice and plated on poly-D-lysine (0.1 mg/ml; Sigma-Aldrich)-coated glass coverslips (30 µg/ml in PBS overnight at 4°C) in Neurobasal-A medium (Invitrogen) supplemented with B-27 serum-free supplement (Invitrogen), 0.5 mM L-glutamine (Invitrogen), 2.5% fetal bovine serum (HyClone, Logan, UT), and 1% penicillin-streptomycin-neomycin (Invitrogen) in ultra violet-sterilized 24-well flat bottom plates (MaxiSorp, Nalge Nunc Int., Naperville, IL). They were then cultured for >10 days at 37°C in a humidified atmosphere of 5% CO_2_ and 95% air. For cultures grown longer than 3 days, the media were replaced with fresh medium on days 3 and 6.

### Immunocytochemistry

For immunocytochemistry experiments, adult NG neuronal cultures (grown for at least 10 days *in vitro*) were fixed in warmed (37°C) 4% paraformaldehyde/30% sucrose mixture, and incubated at room temperature for 10 min. After fixation, the paraformaldehyde/sucrose mixture was aspirated, and 1 ml of 0.1% (vol/vol) Triton X-100 solution in PBS was added and incubated for 10 min at room temperature. Next, the coverslips were washed once gently with 0.1 M PBS, and 1 ml of 5% (wt/vol) bovine serum albumin or 10% serum in PBS was added; samples were then incubated for an hour at room temperature. Primary antibodies (polyclonal rabbit anti P2X1, P2X2, P2X3, and P2X4, or monoclonal mouse anti NeuN; 1∶1000, Cell Signaling Technology) were then diluted in the same solution, added to the coverslips, and incubated overnight in a humidified chamber at 4°C. After rinsing the coverslips three times with PBS, secondary antibodies (goat anti-rabbit fluorescein, goat-anti-mouse Cy3, 1∶1000, Sigma-Aldrich) were added and incubated at room temperature for 1 hour. The cells were then kept in the dark until analysis. Coverslips were rinsed and mounted on slides using Immuno-Fluore mounting medium (ICN Immunobiologicals). The cells were examined by epifluorescence on a Zeiss (Axiovert 35) microscope (Zeiss, Germany).

### Photomicroscopy and Image Analysis

Images of DAB-stained sections were captured using a JVC digital camera (Panasonic, DMC-1, Japan) attached to an Olympus microscope (Olympus, BH-2, Japan). Signals on the NG neurons were analyzed in 10× microscopic visual fields. Signal intensity and the area of each neuron were calculated using the High Vivid Color Pathological Photo Analysis System (HPIAS-1000; Huazhong University of Science and Technology, China) on a Samsung computer. P2X receptor subunit expression in the ganglia was determined by counting all P2X receptor-positive cell profiles in every sixth section throughout the ganglia (approximately eight sections per animal). Cell sizes were determined as described by Rose and Rohrlich [35]. Cell diameters <20 µm were defined as small-diameter neurons, 20–30 µm were medium-diameter neurons, and >30 µm were large-diameter neurons. Counts were made of the number of profiles positive for the P2X receptor in each section and the number of profiles positive for P2X1, P2X2, P2X3, and P2X4 receptor-expressing antigens, and the percentages were calculated. Images of immunofluorescent-labeled cultured cells were captured using a Leica DC 200 digital camera (Leica, Switzerland) attached to a Zeiss Axioplan microscope (Zeiss, Germany). Images were imported into a graphics package (Adobe Photoshop 5.0) Two-channel readings for green and red fluorescence were merged using Image J.

### Whole Cell Patch Clamp Recording and *in Situ* Single Cell Immunostaining

The NG was dissected from 150 g adult rats that had been sacrificed by cervical dislocation. The skull was opened to expose the nodose ganglion in the jugular foramen, which was carefully dissected. The membrane outside the ganglion was torn, and it was then chopped into small pieces using iris scissors. It was then incubated for 30 min at 35°C in Dubecco’s modified Eagle’s medium (DMEM, without Ca^2+^ and Mg^2+^) supplemented with trypsin (type-II-S, 0.5 mg/ml), and collagenase (type I-A, 1 mg/ml; Sigma-Aldrich). The digestion of the cell suspensions was then stopped with 1.25 mg/ml soybean trypsin inhibitor (type II-S, Sigma-Aldrich). The cells were transferred to a culture dish, and left for 30 min before whole cell patch clamp recordings. The cells were placed in the recording dish on the stage of an inverted microscope at room temperature. The media were then replaced with extracellular solution (in mM) (150 NaCl, 5 KCl, 2.5 CaCl_2_, 1 MgCl_2_, 10 HEPES, and 10 D-glucose). The electrodes were pulled using a puller (PUL-2, WPI), and filled with pipette solution (in mM) (150 KCl, 2 MgCl_2_, 10 HEPES, 11 EGTA, and 4 Na_2_ATP). Patch electrodes had a tip resistance of ∼3–5 MΩ. A tight seal was allowed to form between the patch electrode and a cell (resistance ≥1 GΩ), and the membrane was ruptured across the electrode tip to establish the whole-cell patch-clamp configuration. Recordings were then made in the voltage clamp mode with a holding voltage of −60 mV. A tight seal was maintained for more than 5 min before recording. Drugs were applied using a gravity perfusion system. Data were recorded using a LabMaster system, and analyzed with Clampfit 7.0 and Excel. After finishing recording, *in situ* single cell immunostaining was performed. The cell was carefully moved to a gelatin-coated slide, and dried at room temperature for 2 h. The cell was then fixed for 10 min with freshly prepared 4% paraformaldehyde in 0.1 M PBS (0.1 M phosphate, 0.9% w/v saline, pH 7.3), and rinsed in 0.1 M PBS three times for 10 min. The subsequent immunohistochemistry procedures were performed as described above.

### Statistical Analysis

Data are expressed as means ± standard error of the mean (SEM). Student’s *t*-test or analysis of variance (ANOVA) were used, and significance was defined by a *P*-value <0.05.
